# Burdett's Hospital Annual and Year Book of Philanthropy. 1891-1892

**Published:** 1891-12

**Authors:** 


*Burdett's Hospital Annual and Year Book of Philanthropy.* 1891–1892. London: THE HOSPITAL, (LIMITED).

This valuable handbook, in the third year of its publication, is an increasing monument of the indefatigable energy of its author in the collection and compilation of all kinds of statistics which bear upon hospitals, their work and their finance.

English, Scotch, and Irish—general and special—hospitals, and those to which medical schools are attached, are tabulated and compared. We note, by the way, an error which relates to the association of the Bristol Medical School with the Infirmary and General Hospital. Number of patients treated, cost per bed, provisions, alcohol, drugs are recorded for each; and so are salaries of officers, pensions, repairs, and all kinds of incidental expenditure. Detailed tables of analysis allow the reader to use his individual judgment towards any deductions to which the figures may lead him; yet the author makes summaries and draws comparisons, and gives suggestions on all heads with judgment and moderation, such as may be accepted by those who prefer generalisations to particulars.

We think that no responsible hospital official, member of committee, secretary, or matron should be without the *Hospital Annual.* Its contents are of interest to every philanthropist, and the chapters on Hospital Saturday and Sunday funds are full of information as to the collection and distribution of working-men's contributions in aid of hospital expenses. Moreover, the author invites not only co-operation, but suggestions and criticisms upon his statements and opinions. So that there is the prospect of a far better organisation and method for hospital charity than could be possible without the issue of such a fund of information as is here compiled.

Nurses and nursing have for years past exercised the attention of our author, and the two chapters devoted to this subject are well worthy of careful perusal.

The price of the book brings it within the reach of all, and it is destined to play an important part in the proper development of sound hospital administration, whether in town or country.

